# Toughness, elasticity and physical properties for the evaluation of foamed concrete reinforced with hybrid fibers

**DOI:** 10.1016/j.heliyon.2018.e01103

**Published:** 2018-12-27

**Authors:** Eethar Thanon Dawood, Yahya Ziad Mohammad, Waleed Abdulrazzak Abbas, Mohamed Abdul Mannan

**Affiliations:** a Building and Construction Dept., Technical College of Mosul, Northern Technical University, Mosul, Iraq; b Building and Construction Eng., University of Technology, Baghdad, Iraq; c Building and Construction Eng., University of Sarawak, Sarawak, Malaysia

**Keywords:** Civil engineering

## Abstract

This study has been undertaken to investigate the evaluation of Fiber-reinforced Foamed Concrete (FRFC) performance by the use of toughness and non-destructive tests. These tests cover the workability, density, static modulus of elasticity, toughness, ultrasonic pulse velocity and absorption tests. Different FRFC mixes using carbon fibers in the order of 0.5, 1 and 1.5% carbon fibers were used. Also, the combinations of carbon fibers (C) and polypropylene fibers (PP) as 1% C+ 0.5% PP, and 0.5% C+1% PP were prepared. Lastly, the inclusion of polypropylene fibers with the order of 1.5% PP was used to strengthen the foamed concrete mix.

The results showed that the use of 1.5% of C has affected the modulus of elasticity and flexural toughness of foamed concrete.

On the other hand, a strong relationship is found between compressive strength and ultrasonic pulse velocity for FRFC.

## 1 Introduction

Lightweight foamed concretes have been significantly used in many applications of civil engineering areas due to its relatively low cost, beneficial properties of light weight and ease of manufacture and placement, the applications of foamed concrete for lower densities may cover (1) thermal insulation, (2) lightweight blocks and pre-cast panels productions (3) below bituminous finishes for roofs, (4) trench reinstatement, (5) road sub-base, (6) fire insulation, (7) airports and regular traffic shock absorbing barriers and (8) soil stabilization [1, 2].

So far, there are many studies that have been prepared on foamed concrete. Such studies revealed that the foam performance can significantly affect the pore structure of foamed concrete. There is a close relationship between the pore structure of foamed concrete and the properties of foamed concrete such as strength, water absorption and density and modulus of elasticity [3, 4, 5, 6, 7].

Concrete can be classified as a brittle material, the use of discontinuous fibers will influence the strength and toughness. The main factors controlling the fiber reinforced concrete performance are: (1) the mechanical properties of matrix and fibers; (2) the bond strength between matrix and fibers; and (3) the total volume fractions of fibers and their orientation and distribution [8].

The modules and geometrical size of fibers influence the performance of fiber-reinforced concrete (FRC). Thus, the use of suitable type and percentage of fibers enhance the overall mechanical properties of concrete [9].

For lightweight foamed concrete the purpose of fibers use is to reduce non-load cracking of foam concrete at early ages. However, they must have a high modulus of elasticity and be of good strength to improve the required tensile resistance. The fiber reinforcement can change the basic brittleness property of cellular concrete to ductile elasto-plastic materials. Fibers used for production of lightweight foamed concrete are: glass fiber, synthetic fiber and carbon fiber, however, steel fibers are not suitable for foamed concrete since they may settle to the bottom of a concrete mixture [10].

However, most of the recent researches involved with hybrid fibers reinforcement of foamed concrete to boost its performance [9, 10, 11, 12]. Therefore, this paper presents the results of the physical, modulus of elasticity and flexural toughness tests which are powerful techniques for evaluating the mechanical properties of FRFC with regard to their strength and durability apart from assessment and control of the quality of hardened concrete without or with partial damage to the concrete.

## 2 Materials and mix proportions

### 2.1 Materials

Ordinary Portland cement (OPC) which is commercially known as AL-Mass cement factory (Sulaimaniyah governorate of Iraq) was used in this study. The characteristics of ordinary Portland cement are conformed to ASTM C150 [13]. Silica fume was used as a partial replacement of cement. The physical and mechanical properties of cement are presented in Table 1. Whereas, such properties of silica fume are shown in Table 2.

**Table 1:** Physical and mechanical properties of cement.

Properties	Results	Limits of IQS: 5/1984
Initial setting time (minute)	100	≥45 minute
Final setting time (minute)	320	≤600 minute
Fineness (Blaine m^2^/kg)	300	≥230 (m^2^/kg)
Soundness by Autoclave Method (%)	0.02	Not more than 0.8
Compressive strength (MPa)		
3 days	21	≥15
7 days	27	≥23

**Table 2:** Physical and Mechanical Properties of Silica fume.

Properties	Results	ASTM C1240
Specific surface area	15 m^2^/gm	–
Over size particles retained on 45 micron sieve	Less than 10%	34%
Water demand	108%	Less than 115%
Pozzolanic Activity Index, 7 days.	95.6	Not Less than 80%

The natural sand used was supplied from AL- Ukhaider region. The specific gravity and fineness modulus of sand used were 2.65 and 3, respectively. The sieve analysis of aggregate conforms to ASTM C33 [14].

The foaming agent used to get the aerated concrete mix by entraining a controlled amount of air bubbles to concrete mix. The Liquid and Brownish foaming agent used with a specific gravity of 1.01.

Carbon fibers with 8 mm length, 7 μm diameter and density of 1.7 g/cm^3^were used with the lightweight foamed concrete.

Besides, Monofilament polypropylene fibers were included in foamed concrete mixes. Chopped strand shape fibers with a density of 0.9 g/cm^3^, 12 mm long and 18 μm of diameter.

### 2.2 Test methods

The density and absorption, flexural toughness, modulus of elasticity and ultrasonic pulse velocity for all foamed concrete mixes were tested according to ASTM C 642 [15], ASTM C1609 [16], ASTM C469 [17] and BS EN 12504-4 [18], respectively. The average of three specimens was used for all the mentioned tests.

### 2.3 Mix proportions

The different mix proportions of the foamed concrete mixes (C0-C6) were prepared according to ACI 211.2 [19]. Table 3 shows the mix proportions of foamed concrete mixes reinforced with fibers.

**Table 3:** Mix proportions of foamed concrete mixes reinforced with fibers.

Mix type	Mix proportion					C	PP	Flow %
	Cement Kg/m^3^	Sand Kg/m^3^	Water Kg/m^3^	Silica fume Kg/m^3^	Foam Kg/m^3^			
C0	500	1045	210	50	1	–	–	110
C1	500	1045	195	50	1	0.5	–	80
C2	500	1045	205	50	1	1	–	75
C3	500	1045	225	50	1	1.5	–	60
C4	500	1045	210	50	1	1	0.5	80
C5	500	1045	205	50	1	0.5	1	80
C6	500	1045	205	50	1	–	1.5	90

## 3 Results and discussion

### 3.1 Fresh properties of FRFC

The fresh properties of FRFC mixes are given by flow, slump test, and fresh density test results, as depicted in Table 4.

**Table 4:** Flow test, slump test and fresh density for FRFC mixtures.

Mixes	Fibers (%)		Slump test (mm) ASTM C 143 [14]	Fresh density (kg/m^3^) ASTM C 138 [15]
	C	PP		
C0	–	–	Collapse	1820
C1	0.5	–	70	1800
C2	1.0	–	60	1810
C3	1.5	–	50	1820
C4	–	1.5	80	1800
C5	0.5	1.0	65	1800
C6	1.0	0.5	65	1800

#### 3.1.1 Workability

The workability values of FRFC mixes varied among mixes due to volume fraction and types of the fibers as shown in Table 2. The use of 1.5% of carbon fibers (C3) decreases the slump value to 50 mm, while the reference mix had a collapse slump. This can refers to widely decrease in workability of FRFC with the carbon fibers increase [20, 21, 22].

However, for FRFC mixes of polypropylene fibers, the slump value obtained by the inclusion of 1.5% of polypropylene fibers (C4) was 80 mm. The comparison between carbon and polypropylene fibers reveals that the carbon fibers influenced the workability higher than that of polypropylene fibers [23]. Moreover, for FRFC with hybrid fibers, the slump was found to be equal to 65 mm for each hybrid fiber mixes (C5 and C6) either by the addition of 0.5 % carbon fibers (C) plus 1% polypropylene fibers (PP) or 1% C + 0.5% PP.

#### 3.1.2 Fresh density

The fresh densities for all mixes are shown in Table 4. The fresh density of mixes depended on the amount of foam agent. Whilst, the incorporation of fibers did not affect the fresh density of FRFC mixes. However, the fresh density decreases with the inclusion of foam agent compared with normal concrete. This behavior is absolutely attributed to the foam agent in the mix which induces the voids within concrete [21]. On the other hand, the inclusions of fibers either carbon or polypropylene fibers in the foamed concrete mix did not vary the values of the fresh density due to small volumetric percentage of such fibers in the mix and the specific gravity values of the fibers which are as much as that of the control foam concrete mix [22, 23].

### 3.2 Hardened properties of FRFC

#### 3.2.1 Static modulus of elasticity

The results of static modulus of elasticity at 90 days are shown in Table 5.

**Table 5:** Average static modulus of elasticity for FRFC mixes.

Mixes	Fibers (%)		Modulus of elasticity at 90 days (GPa)
	C^a^	PP^b^	
C0	–	–	12.3
C1	0.5	–	13.1
C2	1.0	–	14.9
C3	1.5	–	16.7
C4	–	1.5	12.1
C5	0.5	1.0	13.9
C6	1.0	0.5	15.2

a Refers to carbon fibers.

b Refers to polypropylene fibers.

**Fig. 1:**
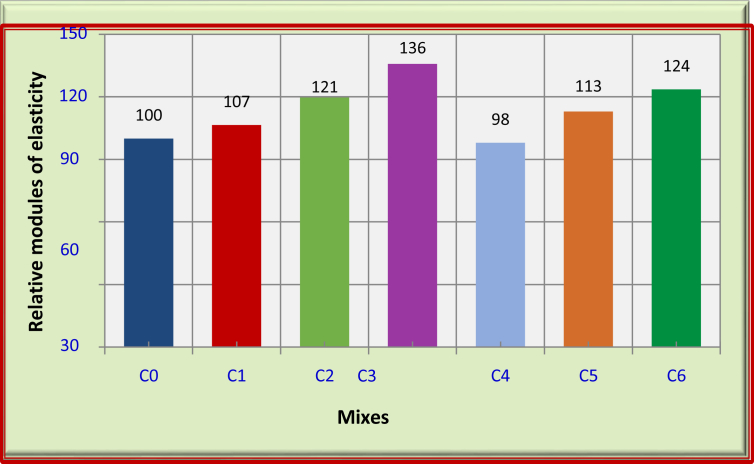
Relative modulus of elasticity for the mixes of FRFC at 90 days.

Generally, the static modulus of elasticity of foamed concrete increases with the compressive strength of foamed concrete increase. It can be noticed that the modulus of elasticity increased with increase in carbon fiber content for the FRFC. Thus, the highest increase in modulus of elasticity of FRFC was recorded with the use of 1.5% carbon fibers (C3) at 90 days. This increase due to such use is around 36% compared with the control mix (C0). While, the incorporation of 0.5% carbon fiber (C1) and 1% carbon fiber (C2) increased the modulus of elasticity by about 7% and 21%, respectively, compared with the control mix as shown Fig. 1. This can be related to the stiffness of the carbon fibers which leads to increase the modulus of elasticity of FRFC [22, 23, 24].

For FRFC reinforced with polypropylene fibers, the modulus of elasticity decreases with polypropylene fibers. The modulus of elasticity decreases by about 2% due to 1.5% PP (C4) compared with the reference mix (C0) as shown in Table 5 and Fig. 1.

For FRFC reinforced with hybrid fibers, the combination of carbon and polypropylene fibers shows good enhancements in modulus of elasticity compared with reference mix (C0). The modulus of elasticity increased by about 13% and 24% due to the combination of 0.5% C + 1% PP (C5) and 1% C + 0.5% PP (C6), respectively, compared with the reference mix.

#### 3.2.2 Flexural toughness

**Fig. 2:**
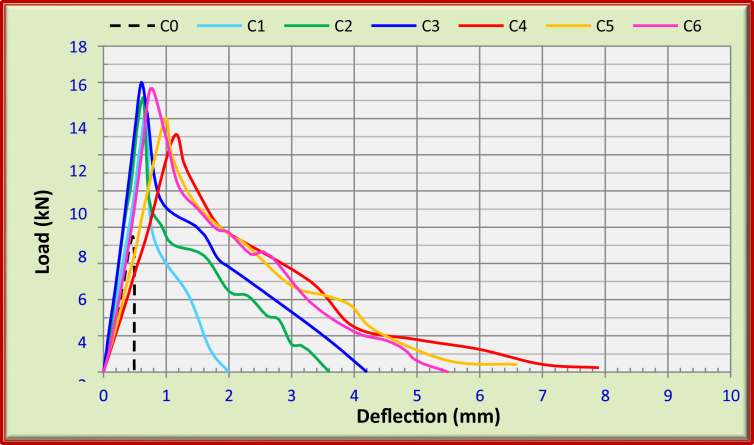
Effect of fibers on flexural toughness of FRFC, using 400*100*100 mm prism.

The flexural toughness results of FRFC at 90 days are shown in Table 6. The results were evaluated according to ASTM C 1609. Fig. 2 shows the relationship between load and deflection of FRFC mixes.

**Table 6:** Average flexural toughness performance for prism 400*100*100 mm, using ASTM C 1609.

Mixes	P_100,0.5_ (kN)	P_100,2.0_ (kN)	f_100,0.5_ (MPa)	f_100,2.0_ (MPa)	T_100,2_ (Joule)
C0	–	–	–	–	–
C1	10.12	0	3.04	0.00	10.46
C2	12.15	4.45	3.65	1.34	14.7
C3	13.45	5.8	4.04	1.74	17.32
C4	5.5	7.65	1.65	2.30	15.92
C5	6.6	7.6	1.98	2.28	16.7
C6	9.25	7.7	2.78	2.31	18.59

For FRFC with carbon fibers, the flexural toughness was enhanced with the carbon fiber increase. From Table 4, it can be noticed that there is a significant increase in the flexural toughness for FRFC with 1.5% carbon fibers (C3) at 90 days.

The parameter of specimen toughness (T_100,2.0_) for mixes C1, C2 and C3 were 10.46, 14.7 and 17.32, respectively, using ASTM C 1609 [10]. Residual loads P_100,0.5_ and P_100,2.0_ for FRFC with 1.5% carbon fibers (C3) were 13.45 and 5.8 kN, respectively.

Such carbon fiber percentage (1.5%) gives the greatest toughness values for mono fibers. This performance is related to the great properties of stiffness and tensile strength of carbon fibers to arrest cracks at both micro- and macro levels. At micro-level, fibers inhibit the initiation of cracks, while at macro-level, fibers donate effective bridging and impart sources of toughness and ductility [23, 24].

For FRFC with polypropylene fibers, the flexural toughness was also improved with polypropylene fibers as shown in Table 6. The parameter of specimen toughness (T_100,2.0_) for mix C4 was 15.92. Besides, the residual loads (P_100,0.5_ and P_100,2.0_) of C4 were 5.5 and 7.65 kN, respectively. For FRFC with hybrid fibers, the combination of carbon and polypropylene fibers boosts the performance of flexural toughness of FRFC mixes. Thus, the mix “C6” showed the greatest values for flexural toughness of FRFC. The parameter of specimen toughness (T_100,2.0_) for mixes “C5 and C6” were 16.7 and 18.59, respectively as shown in Table 4. The residual loads “P_100,0.5_ and P_100,2.0_” of C6 were 9.25 and 7.7 kN, respectively. The greatest flexural toughness value was recorded due to the combination of 1% C + 0.5% PP (C6). The combination of carbon with polypropylene fibers shows the best flexural toughness values compared with individual fibers. The combination enhances the first crack and post crack zones performance of FRFC. However, the carbon fibers have more influence on first crack strength and the polypropylene fibers have more effect on post-crack zone. This behavior is attributed to the stiffness of carbon fibers which are higher than that of polypropylene in a combined fibers system. Thus, reasonable first crack and ultimate strengths are significantly enhanced. Whereas, the enhancements of toughness and strain capacity in the post-crack zone can be obtained due to polypropylene fibers which are relatively flexible [25, 26, 27, 28].

#### 3.2.3 Ultrasonic pulse velocity (UPV)

The results of ultrasonic pulse velocity are shown in Table 7.

**Table 7:** Average ultrasonic pulse velocity for FRFC mixes.

Mixes	Fibers (%)		Ultrasonic pulse velocity at 90 days (m/s)	Compressive strength (MPa) at age 90 days
	C	PP		
C0	–	–	3250	18.4
C1	0.5	–	3340	19.6
C2	1.0	–	3610	24.7
C3	1.5	–	3570	23.8
C4	–	1.5	2850	15.4
C5	0.5	1.0	3420	22.7
C6	1.0	0.5	3530	23.1

**Fig. 3:**
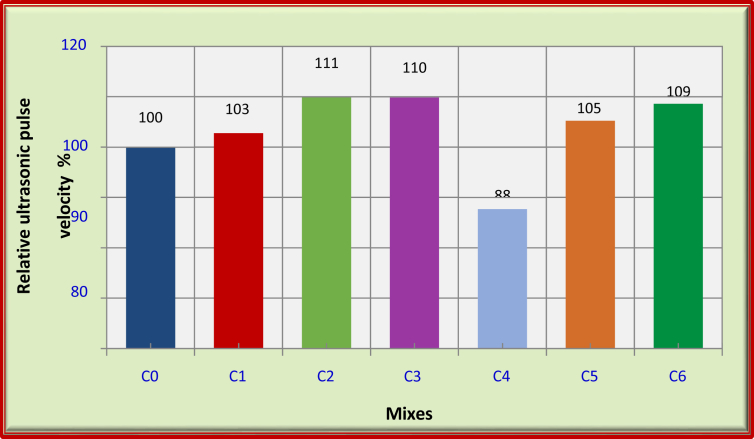
Relative ultrasonic pulse velocity for the mixes of FRFC at 90 days.

For FRFC reinforced with carbon fibers, the use of 0.5% carbon fiber (C1) and 1% carbon fiber (C2) increase UPV by about 3% and 11%, respectively, compared with reference mix (C0). However, further addition of carbon fibers did not improve the UPV. This is because the addition of more fibers initiated the formation of voids by reducing the workability of the mixture. This, in-turn, decreased the speed wave of UPV through the specimen and resulted in a lower UPV value [25] as shown in Table 7 and Fig. 3.

For FRFC with polypropylene fibers, the UPV decreases with the addition 1.5% of polypropylene fibers (C4) by about 12% compared with reference mix (C0). However, it can be observed the increase of UPV of FRFC with hybrid fibers, as shown in Fig. 3.

**Fig. 4:**
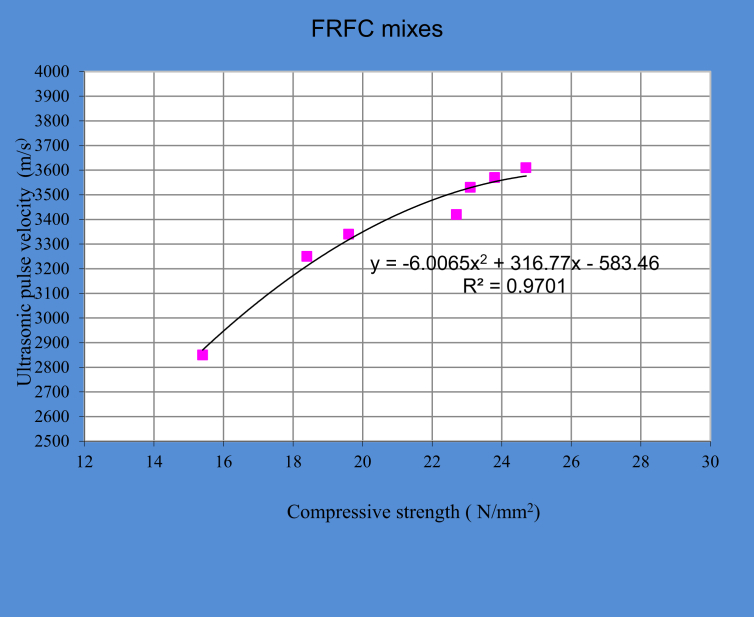
Relationship between compressive strength and ultrasonic pulse velocity of FRFC mixes.

A strong relationship is found between compressive strength and ultrasonic pulse velocity for FRFC mixes as shown in Fig. 4.

#### 3.2.4 Water absorption

The water absorption results for the FRFC are shown in Table 8.

**Table 8:** Average water absorption for FRFC mixes.

Mixes	Fibers (%)		Water absorption at 90 days (%)
	C	PP	
C0	–	–	12.22
C1	0.5	–	9.94
C2	1.0	–	7.52
C3	1.5	–	7.35
C4	–	1.5	8.90
C5	0.5	1.0	8.81
C6	1.0	0.5	9.22

**Fig. 5:**
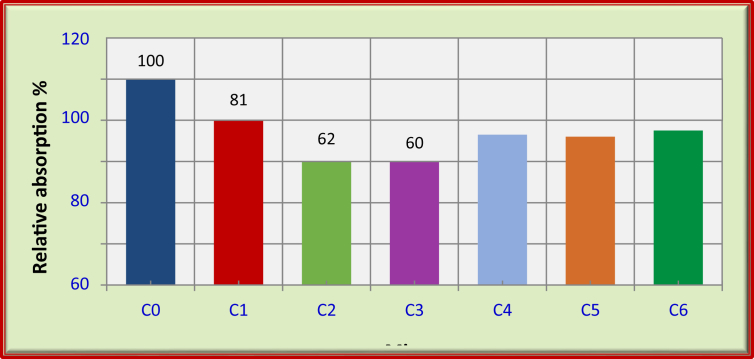
Relative water absorption for the mixes of FRFC at 90 days.

Generally, for FRFC reinforced with carbon fibers, the water absorption decreased by the percentage increase of carbon fibers, as shown in Table 6 and Fig. 5. The reduction in water absorption was about 40% for mix with 1.5% carbon fiber (C3) compared with the control mix (C0). However, the incorporation of 0.5% carbon fiber (C1) and 1% carbon fiber (C2) decreased the water absorption by about 19% and 38%, respectively, compared with the control mix (C0). This behavior is related to the ability of the carbon fibers to reduce the water absorption through the enhancement of the matrix and the interface zone between cement paste and aggregate [28, 29, 30].

For FRFC mixes with polypropylene fibers, the water absorption decreased by about 27% due to the use of 1.5% polypropylene fiber (C4). Whereas the FRFC mixes with hybrid fibers, water absorption decreased by about 28% and 25% due to the combination of 0.5% C + 1% PP (C5) and 1% C + 0.5% PP (C6), respectively, compared with the reference mix (C0).

#### 3.2.5 Oven-dry density

**Fig. 6:**
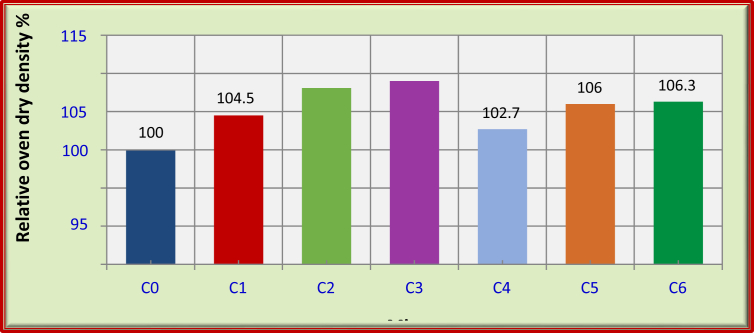
Relative oven dry density for the mixes of FRFC at 90 days.

The results of the oven-dry density test are presented in Table 7. For FRFC mixes with carbon fibers, the oven-dry density increases with the percentage increase of carbon fibers. For FRFC mixes with polypropylene fibers, the oven-dry density somewhat increases with the inclusion of polypropylene fibers. Whereas the FRFC mixes with hybrid fibers oven-dry density increases by the combination of fibers as shown in Table 9 and Fig. 6.

**Table 9:** Average oven dry density for FRFC mixes.

Mixes	Fibers (%)		Oven dry density at 90 days (kg/m^3^)
	C	PP	
C0	–	–	1670
C1	0.5	–	1745
C2	1.0	–	1805
C3	1.5	–	1820
C4	–	1.5	1715
C5	0.5	1.0	1770
C6	1.0	0.5	1775

## 4 Conclusions

The results of toughness and non -destructive tests on FRFC reveal some conclusions as follows:

1. The oven dry density of FRFC increases due to carbon fibers inclusions. However, polypropylene fibers increases slightly the oven dry density due to specific gravity values of such fibers.
2. The inclusion of carbon fibers has significantly affected the modulus of elasticity of FRFC. Thus, the use of 1.5% C exhibits the best performance regarding to such property. The percentage of increase in such use is about 36% higher than that of reference foamed concrete.
3. The flexural toughness of FRFC is significantly enhanced with carbon fibers. However, the combination of carbon with polypropylene fibers boosts the best performance of flexural toughness compared to individual fibers.
4. The absorption of FRFC can be reduced due to fibers used, the dramatic reduction of absorption has been recorded due to 1% and 1.5% of carbon fibers. Thus, the percentages of reduction by such use reach to about 38% and 40% respectively, compared with the reference mix.
5. The addition of 1% of C raised the value of the ultrasonic pulse velocity to 3610 m/s, whereas the control foamed concrete is 3250 m/s. However, a strong relationship is found between compressive strength and ultrasonic pulse velocity for FRFC.

## Declarations

### Author contribution statement

Eethar T. Dawood: Conceived and designed the experiments; Analyzed and interpreted the data; Contributed reagents, materials, analysis tools or data; Wrote the paper.

Yahya Z. Mohammad: Conceived and designed the experiments; Performed the experiments; Analyzed and interpreted the data; Wrote the paper.

Waleed A. Abbas: Conceived and designed the experiments; Wrote the paper.

Mohamed A. Mannan: Analyzed and interpreted the data; Wrote the paper.

### Funding statement

This research did not receive any specific grant from funding agencies in the public, commercial, or not-for-profit sectors.

### Competing interest statement

The authors declare no conflict of interest.

### Additional information

No additional information is available for this paper.
